# Prognostic significance of RNA-based TP53 pathway function among estrogen receptor positive and negative breast cancer cases

**DOI:** 10.1038/s41523-022-00437-7

**Published:** 2022-06-14

**Authors:** Amber N. Hurson, Mustapha Abubakar, Alina M. Hamilton, Kathleen Conway, Katherine A. Hoadley, Michael I. Love, Andrew F. Olshan, Charles M. Perou, Montserrat Garcia-Closas, Melissa A. Troester

**Affiliations:** 1 Department of Epidemiology, University of North Carolina at Chapel Hill, Chapel Hill, NC USA; 2 Division of Cancer Epidemiology and Genetics, National Cancer Institute, Rockville, MD USA; 3 Department of Pathology and Laboratory Medicine, University of North Carolina at Chapel Hill, Chapel Hill, NC USA; 4 Lineberger Comprehensive Cancer Center, University of North Carolina at Chapel Hill, Chapel Hill, NC USA; 5 Department of Genetics, University of North Carolina at Chapel Hill, Chapel Hill, NC USA; 6 Department of Biostatistics, University of North Carolina at Chapel Hill, Chapel Hill, NC USA

**Keywords:** Breast cancer, Prognostic markers, Gene expression, Cancer epidemiology

## Abstract

TP53 and estrogen receptor (ER) are essential in breast cancer development and progression, but TP53 status (by DNA sequencing or protein expression) has been inconsistently associated with survival. We evaluated whether RNA-based TP53 classifiers are related to survival. Participants included 3213 women in the Carolina Breast Cancer Study (CBCS) with invasive breast cancer (stages I–III). Tumors were classified for TP53 status (mutant-like/wildtype-like) using an RNA signature. We used Cox proportional hazards models to estimate covariate-adjusted hazard ratios (HRs) and 95% confidence intervals (CIs) for breast cancer-specific survival (BCSS) among ER- and TP53-defined subtypes. RNA-based results were compared to DNA- and IHC-based TP53 classification, as well as Basal-like versus non-Basal-like subtype. Findings from the diverse (50% Black), population-based CBCS were compared to those from the largely white METABRIC study. RNA-based TP53 mutant-like was associated with BCSS among both ER-negatives and ER-positives (HR (95% CI) = 5.38 (1.84–15.78) and 4.66 (1.79–12.15), respectively). Associations were attenuated when using DNA- or IHC-based TP53 classification. In METABRIC, few ER-negative tumors were TP53-wildtype-like, but TP53 status was a strong predictor of BCSS among ER-positives. In both populations, the effect of TP53 mutant-like status was similar to that for Basal-like subtype. RNA-based measures of TP53 status are strongly associated with BCSS and may have value among ER-negative cancers where few prognostic markers have been robustly validated. Given the role of TP53 in chemotherapeutic response, RNA-based TP53 as a prognostic biomarker could address an unmet need in breast cancer.

## Introduction

TP53 status has generally been observed to be an independent prognostic factor among breast cancer cases^1–6^. However, recent studies suggest the prognostic effect is subtype-dependent, with conflicting reports regarding its prognostic performance^7–10^. It is important to understand the prognostic value of TP53 status within ER subtypes, given that TP53 and ER pathways play essential roles in breast cancer, and due to recent evidence of crosstalk between their signaling pathways^11–14^.

Inconsistent results across previous studies may have been due in part to technical differences. Most studies on TP53 and survival among breast cancer patients have classified TP53 status using either DNA sequencing or immunohistochemistry (IHC) to detect nuclear overexpression of TP53 protein as a surrogate marker of mutation status. IHC methods may misclassify some mutant tumors as wildtype, and both methods may miss some tumors with functional defects in the TP53 pathway^4–16^. In contrast, RNA methods detect patterns of loss or activity downstream in the TP53 signaling pathway. As such, RNA-based TP53 classification methods may reduce misclassification of functional status and clarify associations with survival outcomes. It is also important to address the role of TP53 in in diverse populations and across ER subtypes.

We have sought to address these gaps by evaluating the prognostic value of a validated, RNA-based signature of TP53 functional status (overall and within ER subtypes). Black women have higher rates of TP53 mutant-tumors^15–17^ and may have different mutation types^17^, and therefore, we used data from the Carolina Breast Cancer Study, which oversampled Black and younger women. We compared the prognostic effects of TP53 in this diverse population to those from another large, mostly European dataset.

## Results

The eligible population included 3213 and 1343 breast cancer cases in CBCS and METABRIC, respectively (Table 1, Supplementary Fig. 1). The number of events for each outcome in the two populations are provided in Supplementary Fig. 1. Because the populations differ substantially in the distribution of ER status (50 and 29% ER negative in CBCS and METABRIC, respectively), Table 1 is stratified by ER to facilitate comparisons. Compared to METABRIC, both ER-positive and -negative cases in CBCS were younger at diagnosis, with tumors diagnosed at a lower grade, and a lower proportion of node-positive tumors. As the METABRIC population is predominantly non-Black, the most comparable population is the non-Black subgroup in CBCS. The differences in clinical characteristics between the studies became more pronounced when comparing METABRIC to the non-Black population in CBCS.

**Table 1:** Patient and clinical characteristics, stratified by estrogen receptor (ER) status.

	CBCS		METABRIC	
	ER positive *N* = 2131	ER negative *N* = 1067	ER positive *N* = 1028	ER negative *N* = 303
Median follow-up, years (range)^a^	18.1 (0.2–20.0)	18.0 (0.5–20)	10.0 (0.0–20.0)	7.3 (0.1–20.0)
Median age, years (range)	51 (23–74)	48 (24–74)	63 (26–92)	53 (22–96)
Postmenopausal	1211 (56.8)	516 (48.4)	845 (82.2)	178 (58.7)
Stage				
0/I	968 (45.4)	336 (31.5)	369 (35.9)	79 (26.1)
II	928 (43.5)	564 (52.9)	590 (57.4)	181 (59.7)
III	235 (11.0)	167 (15.7)	68 (6.6)	43 (14.2)
Missing	0	0	1	0
Grade				
1	647 (31.6)	69 (6.5)	109 (10.6)	4 (1.3)
2	896 (42.0)	183 (17.2)	491 (47.8)	28 (9.2)
3	561 (26.3)	815 (76.4)	428 (41.6)	271 (89.4)
Positive node status	810 (38.0)	419 (39.3)	463 (45.0)	163 (53.8)
Tumor size >2 cm	901 (42.3)	625 (58.6)	550 (53.5)	183 (60.4)
RNA-based TP53 status				
Wildtype-like	1038 (75.3)	98 (14.1)	735 (71.5)	23 (7.6)
Mutant-like	340 (24.7)	598 (85.9)	293 (28.5)	280 (92.4)
Missing	753	371	0	0
DNA-based TP53 status				
Wildtype	258 (74.6)	85 (35.3)	775 (77.9)	54 (18.0)
Mutant	88 (25.4)	156 (64.7)	220 (22.1)	246 (82.0)
Missing	1785	826	33	3
IHC-based TP53 status				
Wildtype-like	1531 (76.7)	473 (49.8)	505 (84.4)	92 (53.5)
Mutant-like	465 (23.3)	477 (50.2)	93 (15.6)	80 (46.5)
Missing	135	117	430	131
PAM50 subtype				
Luminal A	885 (64.2)	65 (9.3)	498 (48.6)	11 (3.6)
Luminal B	288 (20.9)	22 (3.2)	325 (31.7)	7 (2.3)
HER2-enriched	67 (4.9)	1.08 (15.5)	61 (6.0)	88 (29.0)
Basal-like	97 (7.0)	465 (66.8)	31 (3.0)	179 (59.1)
Normal-like	41 (3.0)	36 (5.2)	110 (10.7)	18 (5.9)
Missing	753	371	3	0

Figures represent *n* (%) unless otherwise specified. This table excludes *n* = 15 cases from CBCS and *n* = 12 cases from METABRIC that were missing ER status. Individual-level data on race was unavailable in METABRIC.

*CBCS* Carolina Breast Cancer Study, *ER* estrogen receptor, *IHC* immunohistochemistry, *METABRIC* Molecular Taxonomy of Breast Cancer International Consortium, Mut mutant, *PAM50* Prediction Analysis of Microarray 50, *WT* wildtype.

^a^Includes 1315 cases in CBCS (Phases 1-2) and 1331 cases in METABRIC with data on breast cancer-specific survival.

**Fig. 1:**
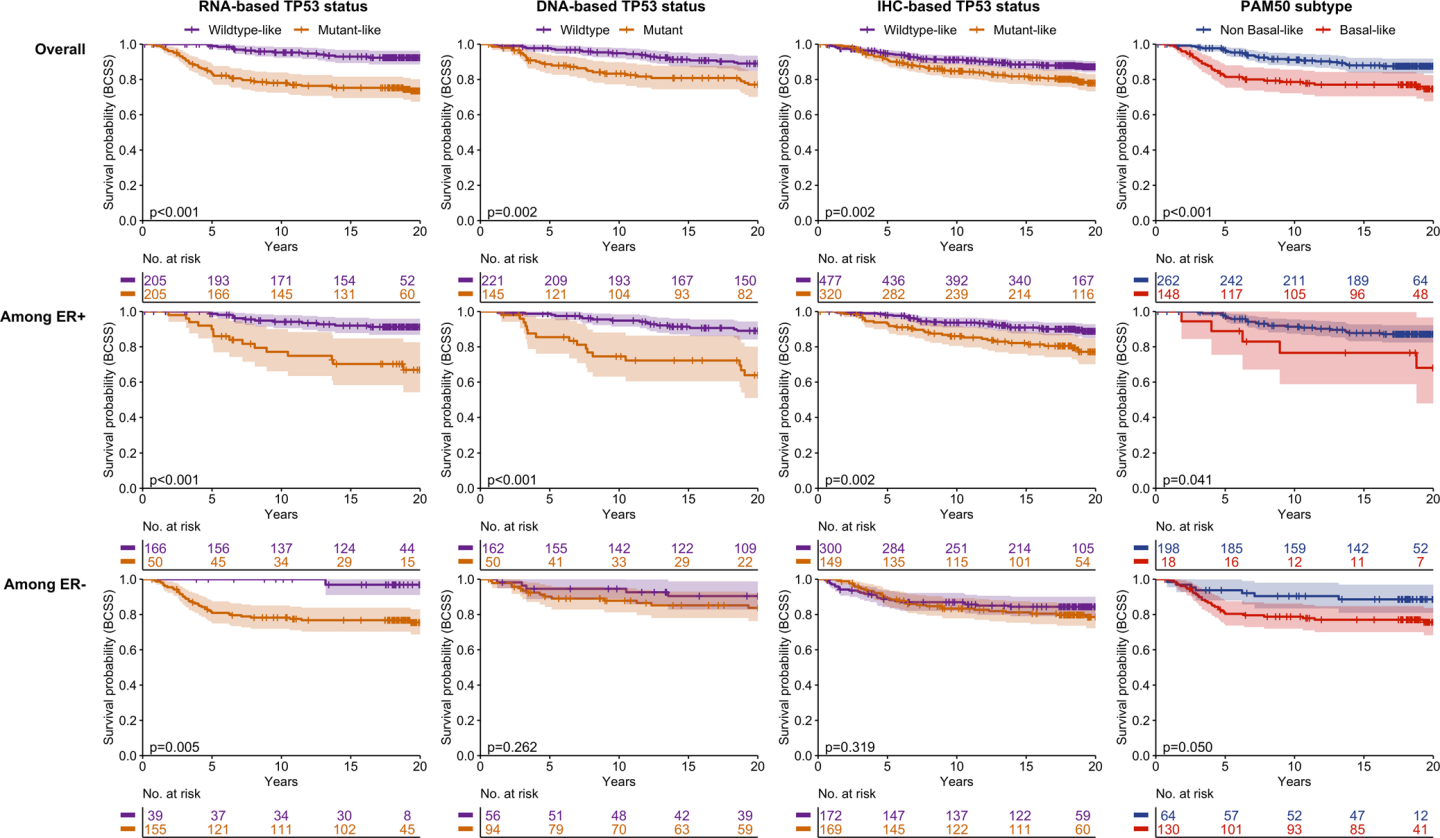
Kaplan–Meier survival curves for breast cancer-specific survival by tumor subtype, overall and stratified by ER status, among node negative breast cancer cases in CBCS. *p* values correspond to the log-rank test. The shaded regions correspond to the 95% confidence interval. BCSS = breast cancer-specific survival, CBCS = Carolina Breast Cancer Study, ER = estrogen receptor, IHC = immunohistochemistry, ER = estrogen receptor, IHC = immunohistochemistry, PAM50 = Prediction Analysis of Microarray 50.

**Fig. 2:**
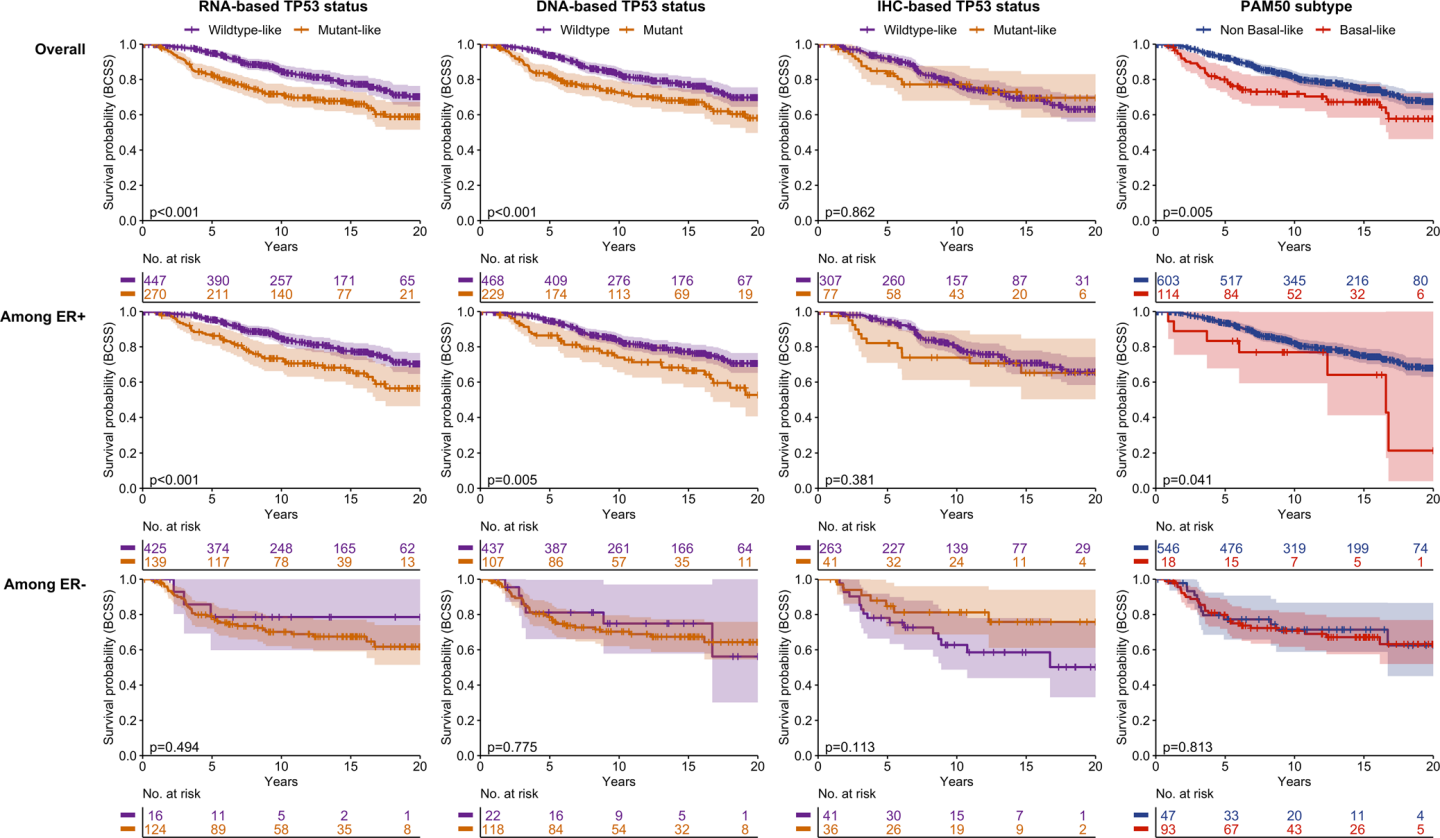
Kaplan-Meier survival curves for breast cancer-specific survival by tumor subtype, overall and stratified by ER status, among node negative breast cancer cases in METABRIC. *p* values correspond to the log-rank test. The shaded regions correspond to the 95% confidence interval. BCSS = breast cancer-specific survival, ER = estrogen receptor, IHC = immunohistochemistry, METABRIC = Molecular Taxonomy of Breast Cancer International Consortium, PAM50 = Prediction Analysis of Microarray 50.

**Fig. 3:**
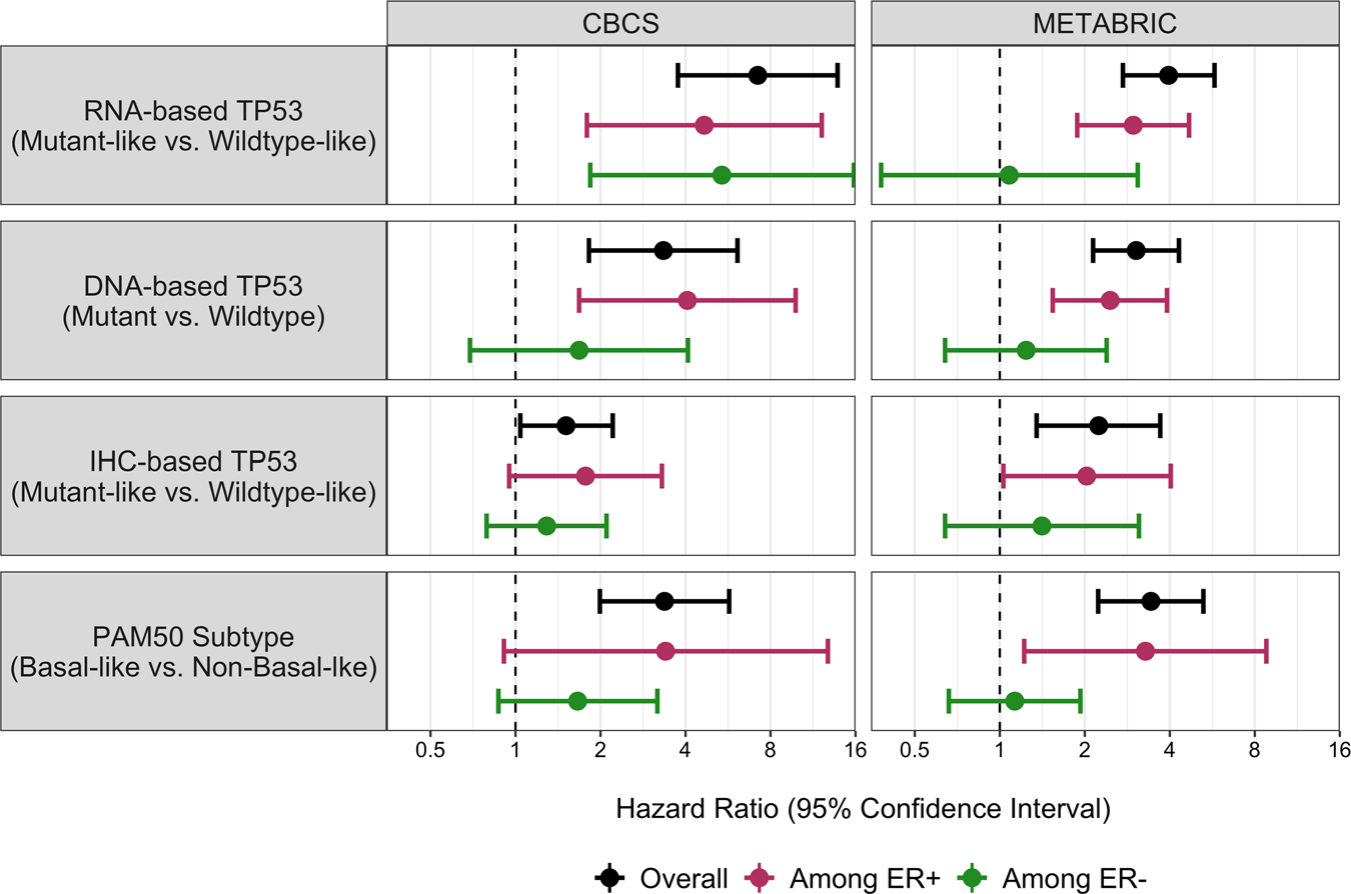
Association between tumor subtype and breast cancer-specific survival among breast cancer cases in CBCS and METABRIC, overall and stratified by estrogen receptor (ER) status. The error bars correspond to the 95% confidence intervals. CBCS = Carolina Breast Cancer Study, ER = estrogen receptor, IHC = immunohistochemistry, METABRIC = Molecular Taxonomy of Breast Cancer International Consortium, PAM50 = Prediction Analysis of Microarray 50.

Breast cancer-specific survival patterns varied across TP53 subtypes. Kaplan Meier plots (Figs. 1, 2) and multivariable models (Tables 2 and 3, Fig. 3) showed that TP53 mutant/mutant-like tumors (by RNA-, IHC-, and DNA-based methods) were associated with worse BCSS compared to wildtype/wildtype-like tumors. The strongest associations were observed for RNA-based TP53 mutant-like status (HR [95% CI] of 7.21 [3.76–13.82] in CBCS and 3.96 [2.73–5.76] in METABRIC), with associations especially attenuated for IHC-based TP53 mutant-like status (1.51 [1.04, 2.21] and 2.24 [1.35, 3.70), respectively). The hazard of TP53 mutant-like status decreased over time, particularly for the RNA-based classification. For example, in CBCS the HR of 7.21 (3.76–13.82) reflects the survival effect of TP53 mutant-like status compared to wildtype-like at one year of follow up, which decreased over time (*T* = 0.81 [0.74–0.87]).

**Table 2:** Hazard ratio (95% confidence interval) for the association between tumor subtype and breast cancer-specific survival among breast cancer cases in CBCS Phases 1–2, overall and stratified by estrogen receptor (ER) status.

Tumor subtype	Overall			ER positive			ER negative		
	*N* (events)	Minimally adjusted^a^	Adjusted^b^	*N* (events)	Minimally adjusted^a^	Adjusted^b^	*N* (events)	Minimally adjusted^a^	Adjusted^b^
RNA-based TP53									
Wildtype-like	340 (59)	Ref.	Ref.	274 (50)	Ref.	Ref.	65 (9)	Ref.	Ref.
Mutant-like	363 (121)	6.94 (3.74–12.87)	7.21 (3.76–13.82)	97 (33)	5.23 (2.09–13.08)	4.66 (1.79–12.15)	265 (88)	4.81 (1.67–13.86)	5.38 (1.84–15.78)
*T*^c^		0.81 (0.75–0.88)	0.81 (0.74–0.87)		0.87 (0.77–0.97)	0.86 (0.77–0.96)		0.86 (0.76–0.99)	0.88 (0.77–0.99)
DNA-based TP53									
Wildtype	347 (69)	Ref.	Ref.	258 (51)	Ref.	Ref.	85 (17)	Ref.	Ref.
Mutant	247 (82)	3.57 (2.00–6.35)	3.34 (1.82–6.11)	88 (31)	4.22 (1.81–9.82)	4.06 (1.68–9.82)	156 (51)	1.81 (0.76–4.31)	1.68 (0.69–4.08)
*T*^c^		0.89 (0.83–0.96)	0.89 (0.83–0.96)		0.89 (0.81–0.99)	0.89 (0.80–0.98)		0.98 (0.86–1.12)	0.99 (0.86–1.13)
IHC-based TP53									
Wildtype-like	752 (156)	Ref.	Ref.	457 (83)	Ref.	Ref.	288 (71)	Ref.	Ref.
Mutant-like	533 (157)	1.66 (1.14–2.41)	1.51 (1.04–2.21)	261 (73)	2.03 (1.10–3.76)	1.77 (0.95–3.30)	267 (84)	1.24 (0.76–2.01)	1.29 (0.79–2.10)
*T*^c^		0.97 (0.93–1.02)	0.97 (0.92–1.02)		0.96 (0.90–1.03)	0.97 (0.90–1.03)		1.01 (0.93–1.09)	1.00 (0.92–1.09)
PAM50 subtype									
Other	473 (104)	Ref.	Ref.	345 (74)	Ref.	Ref.	127 (30)	Ref.	Ref.
Basal-like	230 (76)	3.05 (1.83–5.09)	3.37 (1.99–5.71)	26 (9)	3.62 (1.00–13.12)	3.40 (0.91–12.77)	203 (67)	1.27 (0.67–2.41)	1.66 (0.87–3.18)
*T*^c^		0.86 (0.79–0.93)	0.86 (0.79–0.93)		0.87 (0.73–1.05)	0.87 (0.73–1.05)		1.00 (0.89–1.12)	0.99 (0.88–1.12)

*CBCS* Carolina Breast Cancer Study, *ER* estrogen receptor, *IHC* immunohistochemistry, *PAM50* Prediction Analysis of Microarray 50.

^a^Adjusted for age at diagnosis (continuous), race (Black/non-Black), and study phase.

^b^Additionally adjusted for tumor stage, grade, size, and node status.

^c^Log of time-varying coefficient (if *T* < 1 then hazard decreases with time, and if *T* > 1 then hazard increases with time).

**Table 3:** Hazard ratio (95% confidence interval) for the association between tumor subtype and breast cancer-specific survival among breast cancer cases in METABRIC, overall and stratified by estrogen receptor (ER) status.

Tumor subtype	Overall			ER positive			ER negative		
	*N* (events)	Minimally adjusted^a^	Adjusted^b^	*N* (events)	Minimally adjusted^a^	Adjusted^b^	*N* (events)	Minimally adjusted^a^	Adjusted^b^
RNA-based TP53									
Wildtype-like	764 (189)	Ref.	Ref.	734 (178)	Ref.	Ref.	23 (10)	Ref.	Ref.
Mutant-like	579 (253)	4.95 (3.47–7.06)	3.96 (2.73–5.76)	292 (125)	3.71 (2.38–5.77)	2.97 (1.88–4.68)	280 (126)	1.20 (0.44–3.24)	1.08 (0.38–3.08)
*T*^c^		0.87 (0.83–0.92)	0.87 (0.83–0.91)		0.91 (0.86–0.97)	0.92 (0.87–0.97)		0.93 (0.78–1.12)	0.89 (0.73–1.08)
DNA-based TP53									
Wildtype	838 (234)	Ref.	Ref.	774 (203)	Ref.	Ref.	54 (28)	Ref.	Ref.
Mutant	469 (202)	3.67 (2.61–5.15)	3.04 (2.14–4.31)	219 (95)	3.07 (1.94–4.85)	2.46 (1.54–3.91)	246 (107)	1.10 (0.58–2.08)	1.24 (0.64–2.39)
*T*^c^		0.89 (0.85–0.94)	0.89 (0.85–0.93)		0.94 (0.89–1.00)	0.94 (0.89–1.00)		0.93 (0.83–1.04)	0.91 (0.82–1.02)
IHC-based TP53									
Wildtype-like	600 (178)	Ref.	Ref.	504 (135)	Ref.	Ref.	92 (42)	Ref.	Ref.
Mutant-like	173 (67)	2.87 (1.74–4.71)	2.24 (1.35–3.70)	93 (38)	2.94 (1.50–5.74)	2.03 (1.03–4.03)	80 (29)	1.34 (0.62–2.94)	1.41 (0.64–3.11)
*T*^c^		0.88 (0.81–0.95)	0.88 (0.81–0.96)		0.91 (0.82–1.00)	0.91 (0.83–1.01)		0.88 (0.74–1.04)	0.88 (0.74–1.04)
PAM50 subtype									
Other	1,127 (356)	Ref.	Ref.	992 (287)	Ref.	Ref.	124 (67)	Ref.	Ref.
Basal-like	213 (84)	3.91 (2.57–5.96)	3.43 (2.23–5.26)	31 (14)	3.43 (1.27–9.24)	3.28 (1.22–8.79)	179 (69)	0.99 (0.59–1.66)	1.13 (0.66–1.93)
*T*^c^		0.83 (0.76–0.89)	0.82 (0.76–0.88)		0.91 (0.78–1.06)	0.91 (0.78–1.06)		0.91 (0.82–1.00)	0.90 (0.81–0.99)

*ER* estrogen receptor, *IHC* immunohistochemistry, *METABRIC* Molecular Taxonomy of Breast Cancer International Consortium, *PAM50* Prediction Analysis of Microarray 50.

^a^Adjusted for age at diagnosis (continuous).

^b^Additionally adjusted for tumor stage, grade, size, and node status.

^c^Log of time-varying coefficient (if *T* < 1 then hazard decreases with time, and if *T* > 1 then hazard increases with time).

As 60% of TP53 mutant-like tumors were Basal-like in CBCS, it was of interest to also evaluate Basal-like vs. non-Basal-like subtypes to see whether the survival associations mirrored those for TP53. The Kaplan Meier plots and multivariable models showed that these markers have similar effects. For example, in CBCS the HR (95% CI) for Basal-like vs. non-Basal-like status was 3.37 (1.99–5.71). In multivariable models for both populations, the overall associations were recapitulated when restricting to ER-positive cases. When restricting to ER-negative cases, there were no statistically significant associations between tumor subtypes and BCSS, except in CBCS where the magnitude of association between RNA-based TP53 status and survival was similar among ER-positive and -negative cases (4.66 [1.79–12.15] and 5.38 [1.84–15.78], respectively). Sensitivity analyses restricting CBCS to non-Black cases resulted in no change among ER-positive cases and an increased magnitude among ER-negative cases.

TP53 status was also associated with overall survival, regardless of classification method. Kaplan Meier plots (Supplementary Figs. 2 and 3) only showed statistically significant associations with OS when using DNA-based TP53 classification (as well as RNA-based TP53 in METABRIC). When adjusting for other clinical and tumor characteristics (Supplementary Tables 2 and 3, Supplementary Fig. 4), however, statistically significant associations were observed between all subtype classifications and OS. In CBCS, the strongest associations were observed when using RNA-based TP53 classification, with a similar magnitude among ER-positive and -negative cases. In METABRIC, survival associations were only observed among ER-positive cases.

The association between TP53 status and recurrence-free survival varied by ER status. In both populations, Kaplan Meier plots (Supplementary Figs. 5 and 6) and multivariable models (Supplementary Tables 4 and 5, Supplementary Fig. 7) demonstrated that RNA-based TP53 mutant-like status was associated with worse RFS, but the effect was only observed among ER-positive cases. In CBCS, the association was stronger when using RNA-based TP53 status (6.21 [3.27–11.80]) than when using IHC-based TP53 status (2.16 [1.24–3.78]). In METABRIC, IHC-based TP53 was not associated with RFS.

RNA-based TP53 status provided more prognostic information than the other markers of interest (DNA- and IHC-based TP53, and Basal-like status) in both populations (Supplementary Table 6). Among ER positives, only RNA- and DNA-based TP53 status provided significant prognostic value, with RNA-based TP53 being the greatest contributor (Δ*χ*^2^ [*p* value] = 10.5 [0.005] and 24.7 [<0.001] in CBCS and METABRIC, respectively). Among ER negatives, RNA-based TP53 was the only prognostic marker in CBCS (12.5 [0.002]), and Basal-like status the only prognostic marker in METABRIC (7.5 [0.023]).

It is of interest to understand whether the effects of TP53 status differ between Black and non-Black cases; however, the sample size in CBCS allowed only exploratory analysis of these associations. Among ER-positive cases there were no interactions between RNA- or DNA-based TP53 status and race (*p* = 0.96 and 0.78, respectively), but an interaction was observed by IHC-based TP53 status (*p* = 0.03). Specifically, the association between mutant-like status and poorer BCSS was more pronounced for non-Black cases compared to Black cases. Among ER-negative cases there were suggestions of interactions between RNA- and DNA-based TP53 and race (*p* = 0.18 and 0.12, respectively), with the association between TP53 mutant/mutant-like status and poorer BCSS being more pronounced for non-Black cases compared to Black cases. No interaction, however, was observed when using IHC-based TP53 status (*p* = 0.52).

## Discussion

RNA-based TP53 functional score had stronger prognostic value than other technical methods in a population-based cohort including Black and Non-Black women in North Carolina. The survival effect of TP53 mutant-like status was most consistent among ER-positive cases, but also showed significant effects among ER-negative cases in CBCS (where ER negatives were prevalent at 33%). Given the proportion of cases who had both TP53 mutant-like and Basal-like phenotypes, it was important to also evaluate the effects of TP53 among Basal-like vs. non-Basal-like. The BCSS associations for Basal-like and TP53 were similar, but more high-risk cases were captured with the TP53 status classification. TP53 is an important prognostic marker with potential clinical value and may be useful among ER-negative patients for whom prognostic markers are otherwise lacking.

Prior studies have evaluated the survival effects of IHC and DNA-based TP53 status among breast cancer patients, with near consensus that TP53 mutant cases have poorer survival compared to wildtype (Table 4)^1–4,6,7,18,19^. Very few studies, however, have assessed survival differences by ER status. Among those that have, TP53 mutant cases were generally associated with worse outcomes among ER-positive cases^7,9,10,20^, in line with our findings. However, results among ER-negatives have been more mixed, with some reporting TP53 mutant cases having better survival^9^, but most finding no effect^7,10,21^. It may seem paradoxical that the more aggressive tumors were sometimes found to have better outcomes, but several mechanisms have been proposed, largely indicating enhanced chemosensitivity in ER negative/TP53 mutant tumors. In the present study we found a strong association between TP53 mutant-like status and poorer BCSS among ER negatives, which may demonstrate the importance of functional TP53 status over other classification methods. Additionally, the present findings come from a population-based study, unlike all other previous studies.

**Table 4:** Previously published associations between TP53 status and overall survival, by estrogen receptor (ER) status and TP53 classification method.

IHC				DNA				RNA			
		Effect of TP53 mut-like				Effect of TP53 mut				Effect of TP53 mut-like	
Publication	N cases (TP53 mut-like)	Estimate	Direction	Publication	N cases (TP53 mut)	Estimate	Direction	Publication	N cases (TP53 mut-like)	Estimate	Direction
Overall											
Rossner^38^	859 (307)	0.80 (0.47, 1.33)^a^	X	Shiao^41^	47 (9)	0.64 (0.07, 5.51)	X				
Coates^9^	1113 (303)	1.12 (0.89, 1.39)	X	Rossner^38^	859 (128)	1.04 (0.59, 1.85)^a^	X				
Yamashita^39^	73 (16)	2.36 (1.20, 4.67)	↓↓	Andersson^42^	370 (105)	1.33 (0.92, 1.93)	↓				
Song^4^	440 (227)	3.10 (1.02, 9.44)	↓↓	Powell^5^	1037 (178)	1.9 (1.3, 2.8)	↓↓				
Iwaya^40^	31 (5)	*p* < 0.01	↓↓	Pharoah^1^	2319 (539)	2.0 (1.7, 2.5)	↓↓				
Blaszyk^6^	90 (32)	*p* = 0.0001	↓↓	Silwal-Pandit^7^	1420 (402)	2.03 (1.65, 2.48)^a^	↓↓				
				Olivier^43^	1107 (144)	2.40 (1.70, 3.38)^b^	↓↓				
				Dobes^2^	204 (54)	5.38 (2.14, 13.52)	↓↓				
				Shiao^41^	45 (9)	5.62 (1.37, 23.00)^c^	↓↓				
				Bergh^44^	297 (65)	p = 0.02^a^	↓↓				
				Meric-Bernstam^3^,	165 (47)	*p* = 0.0004	↓↓				
				Ungerleider^45^	1979 (663)	*p* < 0.0001	↓↓				
ER positive											
Coates^9^	880 (171)	1.29 (0.98, 1.70)	↓	Caleffi^21^	106 (17)	*p* = 0.37	X	Coutant^10^	134 (49)	2.43 (0.96, 6.15)	X
Feeley^20^	359 (48)	1.96 (1.00, 3.84)	↓↓	Silwal-Pandit^7^	1037 (195)	1.86 (1.39, 2.49)^a^	↓↓	Coutant^10^	191 (101)	2.30 (1.25, 423)^d^	↓↓
ER negative											
Coates^9^	212 (125)	0.62 (0.40, 0.97)	↑↑	Silwal-Pandit^7^	306 (196)	1.15 (0.77, 1.72)^a^	X	Coutant^10^	64 (41)	*p* = 0.27	X
				Caleffi^21^	86 (26)	*p* = 0.87	X				

Double downward arrows represent a significant decreased risk of survival. A single downward arrow represents a non-significant decreased risk of survival. X’s represent a null association with survival. Double upward arrows represent a significant increased risk of survival.

*ER* estrogen receptor, *IHC* immunohistochemistry, *mut* mutant.

^a^Breast cancer specific survival.

^b^Among PR positives.

^c^Among Black cases.

^d^Distant metastasis-free survival.

Sampling differences between METABRIC and CBCS may explain differences in results among ER-negative cases. In METABRIC, the sample size of ER-negative cases was relatively small (*n* = 303) and among these, almost all (92%) were classified as TP53 mutant-like by the RNA signature. Whereas in CBCS, there was a larger sample of ER-negatives (*n* = 1067), which included a smaller proportion TP53 mutant-like cases (86%). CBCS ER-negative cases were also lower grade and more frequently node negative. Given that the METABRIC samples were sourced from tumor banks, it is plausible that this study oversampled more aggressive tumors, reducing variation of TP53 phenotypes. It is also possible that the more diverse CBCS population led to a different distribution of TP53 mutations (i.e., different types of mutations). Ethnically diverse population-based studies incorporating multigene signatures are important for understanding the diversity of ER negative cases. When population characteristics become a key consideration in interpreting differences across studies, it suggests that either selection bias or relevant variables that vary across populations have not been addressed. However, the current study does show that stratification by ER status is critical and should be included in future studies of TP53-based prognostication.

A strength of this analysis was the racially diverse population with more younger women, and a larger proportion of ER-negative cases. Previous studies of TP53 and prognosis have included populations that are exclusively, or nearly exclusively, of European descent. Another strength was availability of data on TP53 status using three different classification methods. Perhaps the most important limitation was that we did not model treatment differences, precluding the assessment of the predictive value of TP53. A lesser limitation was our choice to use the full dataset for each classification method, inhibiting comparability across methods; but sensitivity analysis in METABRIC among those with complete data for all three classification methods (*n* = 752) produced effect estimates that were unchanged or slightly stronger than those reported in the main analysis. Due to overlap of the TP53 mutant-like and Basal-like phenotypes, we evaluated survival effects of Basal-like vs. non-Basal-like, but we did not evaluate all possible comparisons (e.g., Basal-like versus each of the other individual PAM50 intrinsic subtypes) because even within relatively large data sets, sample sizes did not allow for further stratification. Lastly, this RNA-based TP53 signature has been widely used and validated for research purposes and is operationalized using cohort normalization. However, a single sample predictor has not yet been developed, so it cannot be applied to a single sample or small cohort without making important assumptions. If this signature continues to demonstrate clinical value, development of a single sample is warranted.

The science of prognostication and prediction has generally been led by applications for ER-positive cases and has relied on factors that reflect tumor growth (e.g., proliferation scores). A marker such as TP53, which represents underlying tumor biology and may define molecular vulnerabilities to chemotherapeutics^22,23^, could address an unmet need. Particularly as immunotherapies become widely utilized, markers that identify tumors likely to benefit will be important. Homologous recombination deficiency status has been proposed as one possible approach^24,25^, but TP53 status may also merit consideration. RNA-based TP53 may be particularly valuable because of its interpretability as a pathway-level change and because it can be conveniently paired with other RNA-based assays. Further consideration of multigene TP53 scores in clinical care could be particularly important for ER-negative cases, for whom fewer predictive biomarkers are currently available.

## Methods

### Study populations

The Carolina Breast Cancer Study (CBCS) is a population-based study that enrolled participants in three phases between 1993 and 2013. Study details have been described previously^26^. Briefly, incident invasive breast cancers among women 20–74 years of age were identified using rapid case ascertainment. Black women and those younger than 50 years of age were oversampled. Clinical characteristics at diagnosis were assessed by collecting medical records and formalin-fixed paraffin-embedded (FFPE) tumor samples at study enrollment. All CBCS study procedures were approved by the University of North Carolina School of Medicine Institutional Review Board and participants provided written informed consent.

We compared the results from CBCS to those from the Molecular Taxonomy of Breast Cancer International Consortium (METABRIC), which includes fresh-frozen primary breast tumors collected from five tumor banks across UK and Canada between 1977 and 2005. Clinical and genomic data was downloaded from cbioportal (http://www.cbioportal.org/study?id=brca_metabric). About 93% of subjects were of European descent and the population ranges in age at diagnosis from 22 to 96 years. With an age distribution that skews older (median = 61 years), METABRIC includes a large proportion of ER-positive cases (77%).

Eligible cases were those diagnosed at stage I–III, with available data on TP53 status (Supplementary Fig. 1). In METABRIC, only cases with data on tumor characteristics (stage, grade, size, and node status) were included.

### Breast tumor markers

#### CBCS

ER status was abstracted from clinical records for Phases 1–2. When missing, ER status was determined by the UNC central laboratory. For Phase 3, ER status for all cases was determined by the central laboratory. Concordance between central laboratory and clinical record was 93%^27^. Methods for tissue processing and IHC analysis of tumor markers have been described previously^17,27–29^. ER positivity and TP53 mutant-like status was defined using a 10% positivity threshold. We selected the 10% cutoff for ER because at the time of enrollment for Phases 1–2, it was not yet the clinical standard to classify ER borderline tumors (1% to <10% positivity) as ER positive. Additionally, a 10% cutoff for ER positivity has been shown to have a stronger association with molecular phenotypes (e.g., intrinsic subtypes)^27^. Tumor stage and size were abstracted from the medical records. Tumor grade was defined by centralized pathology review.

RNA expression in CBCS has been quantified using NanoString assays on at least one FFPE tumor sample per patient, with random replication to assess reproducibility^27,30,31^. A previously validated RNA signature that aggregates expression information on TP53-dependent genes was used to classify TP53 functional status (mutant-like or wildtype-like) based on a similarity-to-centroid approach (Supplementary Table 1)^32^. A research version of the PAM50 predictor was used to classify tumors into intrinsic subtypes^30,33^, which were then dichotomized as basal-like or non-basal-like (i.e., luminal A, luminal B, HER2-enriched, or normal-like).

For cases in CBCS phase 1, two complementary DNA-based methods were employed for detecting TP53 mutations using FFPE tumor samples. First, single strand conformational polymorphism (SSCP) analysis was used as a screening procedure to detect mutations in exons 4–8 of the TP53 gene, with subsequent manual radiolabeled sequencing of SSCP positives^34^. The Roche p53 Amplichip research test was also used to detect single base pair substitutions and single base pair deletions in exons 2–11, as well as splice sites (2 base pairs before and after each exon), in the TP53 gene^35^. All assays were carried out by the UNC central laboratory.

#### METABRIC

ER status, as well as other tumor characteristics (tumor grade, stage, and size) were obtained from the medical records. RNA and DNA were extracted for transcriptional and genomic profiling on the Illumina Human v3 microarray and Affymetrix SNP 6.0 platforms, respectively^36^. Tumors were classified for TP53 functional status (mutant-like/wildtype-like) using the RNA-based TP53 signature^32^ and for PAM50 intrinsic subtype (basal-like/non-basal-like) using a research version of the PAM50 predictor^30,33^.

### Outcome assessment

The follow-up period for both studies is defined as the number of years between diagnosis and breast cancer death (for breast cancer-specific survival (BCSS)) and death due to any cause (for overall survival (OS)). For CBCS Phases 1–2, vital status and date of death were determined by linking with the National Death Index (NDI) in 2020. Breast cancer deaths were defined using the International Classification of Diseases breast cancer codes 174.9 (ICD-9) or C50.9 (ICD-10) as derived from death certificates. For METABRIC, vital status and time to death were obtained from the medical records.

Recurrence-free survival (RFS) was defined as time in years from diagnosis to first subsequent recurrent breast cancer (either local, regional, or distant). In CBCS Phase 3, recurrence date was abstracted from medical records after a patient reported a recurrence during follow-up telephone interviews (occurring at regular intervals). In METABRIC, recurrences and time to recurrences were obtained from the medical records.

All subjects who did not experience the outcome of interest were administratively censored at their date of last contact or the last linkage date to the NDI (for CBCS).

### Statistical analyses

Kaplan-Meier plots were generated to compare survival patterns between TP53 subtypes defined using different classification methods (RNA signature, DNA sequencing, and IHC). Because of the overlap in TP53 mutant status and Basal-like intrinsic subtype, we also evaluated survival patterns by PAM50 intrinsic subtype (Basal-like/non-Basal-like) to determine whether the effects mirrored those for TP53. Survival patterns were assessed overall and within ER subtypes. Differences between the curves were evaluated using log-rank tests. Kaplan-Meier plots were restricted to node negative cases, while in multivariable models we retained these cases and included node status as an adjustment factor.

The prognostic value of the TP53 subtypes was evaluated using Cox proportional hazards models to compute hazard ratios (HRs) and 95% confidence intervals (CIs), overall and stratified by ER status, analyzing each TP53 classification method (RNA-, IHC, and DNA-based) separately. Again, we estimated survival effects for PAM50 intrinsic subtype (Basal-like vs. non-Basal-like) to assess whether they mirrored those for TP53. Minimally adjusted models accounted for age at diagnosis (as well as race and study phase in CBCS). Fully adjusted models additionally accounted for tumor stage, grade, size, and node status. Since tumor grade was missing for about 26% of cases in CBCS, covariates with missing values were addressed using the multiple imputation plus outcome approach^37^. TP53 status and PAM50 subtype were modeled with addition of a time-varying term (*T*) due to the observed violation of the proportionality assumption of the Cox model. The direction and magnitude of the change in HR over time is indicated by the log of this coefficient (i.e., log(*T)* < 1 indicates a decreasing hazard and log(*T*) > 1 indicates an increasing hazard). We estimated the prognostic value of each TP53 classification method as the change in likelihood ratio chi square (Δ*χ*^2^) following a likelihood ratio test that compares the full prognostic model to a model after removing each TP53 classification schema. All statistical tests were two-sided and *p* value < 0.05 was used as the cut point for statistical significance. Statistical analyses were conducted in R software version 4.0.2 (R Foundation for Statistical Computing).

### Reporting summary

Further information on research design is available in the Nature Research Reporting Summary linked to this article.

## Supplementary information

Supplementary Tables and Figures

Reporting Summary

## Data Availability

The CBCS datasets generated and/or analyzed during the current study are not publicly available due to some human subjects restrictions, but may be available from the corresponding author on reasonable request. METABRIC data can be found here: http://www.cbioportal.org/study?id=brca_metabric.
