# Supplementary material for: Prognostic significance of RNA-based TP53 pathway function among estrogen receptor positive and negative breast cancer cases
Source: NPJ Breast Cancer. 2022 Jun 14;8:74. doi: 10.1038/s41523-022-00437-7 (PMC9198049; doi:10.1038/s41523-022-00437-7)
Supplement: Supplementary file 1 — Supplementary Tables and Figures [file 41523_2022_437_MOESM1_ESM.pdf]

Supplementary Table 1. Genes included in the TP53 expression signature.

Gene	Entrez	Direction[a]
APH1B	83464	-1
ATAD2	29028	1
AURKA	6790	1
BTG2	7832	-1
C21orf45	54069	1
CCNA2	890	1
CCND1	595	-1
CDC25B	994	1
CDC25C	995	1
CDCA7L	55536	1
CDK1	983	1
CDKN1A	1026	-1
CDKN3	1033	1
CENPF	1063	1
CEP55	55165	1
CKS1B	1163	1
DDB2	1643	-1
FAM198B	51313	-1
FNBP1	23048	-1
FOXM1	2305	1
GATA3	2625	-1
GGH	8836	1
KIAA0040	9674	-1
KIAA1370	56204	-1
KIF23	9493	1
KIFC1	3833	1
LOC400043	400043	-1
MAD2L1	4085	1
MAP2K4	6416	-1
MCM3	4172	1
MKI67	4288	1
MYBL2	4605	1
NCAPH2	29781	1
NEO1	4756	-1
NPEPPS	9520	-1
NUDT1	4521	1
POLD1	5424	1
PREP	5550	1
PTTG1	9232	1
RFC4	5984	1
RNF103	7844	-1
SLC39A6	25800	-1
TAP1	6890	1
TCEAL1	9338	-1
TOP2A	7153	1
TRIP13	9319	1
TUBA4A	7277	1
UBE2C	11065	1

[a]The values of -1 or 1 refer to whether the gene is downregulated or upregulated, respectively, in the expression signature for mutant-like tumors

Supplementary Table 2. Hazard ratio (95% confidence interval) for the association between tumor subtype and **overall survival** among breast cancer cases in **CBCS** phases 1-2, overall and stratified by estrogen receptor (ER) status.

	**Overall**			**ER positive**			**ER negative**		
Tumor subtype	N (events)	Minimally adjusted[a]	Adjusted[b]	N (events)	Minimally adjusted[a]	Adjusted[b]	N (events)	Minimally adjusted[a]	Adjusted[b]
**RNA-based TP53**									
Wildtype-like	340 (145)	Ref.	Ref.	274 (118)	Ref.	Ref.	65 (27)	Ref.	Ref.
Mutant-like	363 (166)	4.77 (3.05 to 7.44)	5.87 (3.59 to 9.59)	97 (49)	3.95 (2.01 to 7.75)	4.14 (2.01 to 8.52)	265 (117)	3.21 (1.51 to 6.83)	4.59 (2.05 to 10.27)
T[c]		0.85 (0.81 to 0.89)	0.85 (0.81 to 0.89)		0.90 (0.84 to 0.96)	0.89 (0.83 to 0.96)		0.87 (0.80 to 0.94)	0.87 (0.80 to 0.94)
**DNA-based TP53**									
Wildtype	347 (152)	Ref.	Ref.	258 (116)	Ref.	Ref.	85 (35)	Ref.	Ref.
Mutant	247 (131)	3.71 (2.37 to 5.82)	3.64 (2.26 to 5.85)	88 (52)	3.18 (1.67 to 6.03)	2.98 (1.53 to 5.82)	156 (78)	2.96 (1.43 to 6.16)	2.98 (1.39 to 6.38)
T[c]		0.91 (0.87 to 0.95)	0.90 (0.86 to 0.95)		0.94 (0.89 to 1.01)	0.94 (0.88 to 1.00)		0.90 (0.84 to 0.98)	0.91 (0.84 to 0.98)
**IHC-based TP53**									
Wildtype-like	752 (328)	Ref.	Ref.	457 (204)	Ref.	Ref.	288 (122)	Ref.	Ref.
Mutant-like	533 (254)	1.74 (1.29 to 2.35)	1.69 (1.25 to 2.30)	261 (131)	1.94 (1.23 to 3.06)	1.72 (1.08 to 2.74)	267 (122)	1.31 (0.87 to 1.97)	1.42 (0.94 to 2.14)
T[c]		0.96 (0.93 to 0.99)	0.96 (0.93 to 0.99)		0.96 (0.92 to 1.01)	0.96 (0.92 to 1.01)		0.97 (0.93 to 1.02)	0.97 (0.92 to 1.01)
**PAM50 subtype**									
Other	473 (207)	Ref.	Ref.	345 (153)	Ref.	Ref.	127 (54)	Ref.	Ref.
Basal-like	230 (104)	2.89 (1.91 to 4.39)	3.39 (2.18 to 5.27)	26 (14)	2.83 (1.02 to 7.84)	2.77 (0.98 to 7.84)	203 (90)	1.40 (0.82 to 2.38)	1.97 (1.13 to 3.43)
T[c]		0.88 (0.84 to 0.93)	0.88 (0.84 to 0.93)		0.92 (0.82 to 1.03)	0.92 (0.82 to 1.04)		0.95 (0.89 to 1.02)	0.95 (0.89 to 1.01)

[a]Adjusted for age at diagnosis (continuous), race (Black/non-Black), and study phase

[b]Additionally adjusted for tumor stage, grade, size, and node status

[c]Log of time-varying coefficient (if $T < 1$ then hazard decreases with time, and if $T > 1$ then hazard increases with time)

CBCS = Carolina Breast Cancer Study, ER = estrogen receptor, IHC = immunohistochemistry, PAM50 = Prediction Analysis of Microarray 50.

Supplementary Table 3. Hazard ratio (95% confidence interval) for the association between tumor subtype and **overall survival** among breast cancer cases in **METABRIC**, overall and stratified by estrogen receptor (ER) status.

	Overall			ER positive			ER negative		
Tumor subtype	N (events)	Minimally adjusted[a]	Adjusted[b]	N (events)	Minimally adjusted[a]	Adjusted[b]	N (events)	Minimally adjusted[a]	Adjusted[b]
**RNA-based TP53**									
Wildtype-like	764 (382)	Ref.	Ref.	734 (363)	Ref.	Ref.	23 (15)	Ref.	Ref.
Mutant-like	579 (335)	3.71 (2.80 to 4.92)	3.27 (2.44 to 4.39)	292 (176)	2.68 (1.88 to 3.81)	2.39 (1.67 to 3.43)	280 (155)	1.16 (0.50 to 2.66)	1.05 (0.44 to 2.48)
T[c]		0.88 (0.86 to 0.91)	0.88 (0.85 to 0.91)		0.92 (0.88 to 0.96)	0.92 (0.89 to 0.96)		0.93 (0.82 to 1.05)	0.90 (0.79 to 1.03)
**DNA-based TP53**									
Wildtype	838 (432)	Ref.	Ref.	774 (394)	Ref.	Ref.	54 (33)	Ref.	Ref.
Mutant	469 (274)	3.45 (2.60 to 4.56)	3.17 (2.38 to 4.24)	219 (136)	2.70 (1.86 to 3.92)	2.43 (1.66 to 3.55)	246 (135)	1.15 (0.63 to 2.10)	1.28 (0.69 to 2.38)
T[c]		0.89 (0.86 to 0.92)	0.89 (0.86 to 0.92)		0.93 (0.89 to 0.97)	0.93 (0.89 to 0.97)		0.94 (0.86 to 1.03)	0.93 (0.85 to 1.01)
**IHC-based TP53**									
Wildtype-like	600 (299)	Ref.	Ref.	504 (247)	Ref.	Ref.	92 (50)	Ref.	Ref.
Mutant-like	173 (93)	2.28 (1.52 to 3.42)	2.00 (1.32 to 3.03)	93 (53)	1.75 (1.01 to 3.04)	1.44 (0.82 to 2.53)	80 (40)	1.45 (0.74 to 2.82)	1.51 (0.77 to 2.96)
T[c]		0.92 (0.87 to 0.96)	0.92 (0.87 to 0.97)		0.95 (0.89 to 1.01)	0.95 (0.89 to 1.02)		0.92 (0.83 to 1.03)	0.93 (0.83 to 1.03)
**PAM50 subtype**									
Other	1,127 (606)	Ref.	Ref.	992 (520)	Ref.	Ref.	124 (79)	Ref.	Ref.
Basal-like	213 (109)	3.60 (2.52 to 5.14)	3.50 (2.43 to 5.02)	31 (17)	2.63 (1.08 to 6.41)	2.66 (1.09 to 6.52)	179 (91)	0.86 (0.54 to 1.37)	0.94 (0.58 to 1.51)
T[c]		0.85 (0.81 to 0.90)	0.85 (0.80 to 0.89)		0.90 (0.80 to 1.02)	0.91 (0.80 to 1.02)		0.97 (0.90 to 1.04)	0.97 (0.90 to 1.04)

[a]Adjusted for age at diagnosis (continuous)

[b]Additionally adjusted for tumor stage, grade, size, and node status

[c]Log of time-varying coefficient (if $T < 1$ then hazard decreases with time, and if $T > 1$ then hazard increases with time)

ER = estrogen receptor, IHC = immunohistochemistry, METABRIC = Molecular Taxonomy of Breast Cancer International Consortium, PAM50 = Prediction Analysis of Microarray 50.

Supplementary Table 3. Hazard ratio (95% confidence interval) for the association between tumor subtype and **overall survival** among breast cancer cases in **METABRIC**, overall and stratified by estrogen receptor (ER) status.

	**Overall**			**ER positive**			**ER negative**		
Tumor subtype	N (events)	Minimally adjusted[a]	Adjusted[b]	N (events)	Minimally adjusted[a]	Adjusted[b]	N (events)	Minimally adjusted[a]	Adjusted[b]
**RNA-based TP53**									
Wildtype-like	764 (382)	Ref.	Ref.	734 (363)	Ref.	Ref.	23 (15)	Ref.	Ref.
Mutant-like	579 (335)	3.71 (2.80 to 4.92)	3.27 (2.44 to 4.39)	292 (176)	2.68 (1.88 to 3.81)	2.39 (1.67 to 3.43)	280 (155)	1.16 (0.50 to 2.66)	1.05 (0.44 to 2.48)
T[c]		0.88 (0.86 to 0.91)	0.88 (0.85 to 0.91)		0.92 (0.88 to 0.96)	0.92 (0.89 to 0.96)		0.93 (0.82 to 1.05)	0.90 (0.79 to 1.03)
**DNA-based TP53**									
Wildtype	838 (432)	Ref.	Ref.	774 (394)	Ref.	Ref.	54 (33)	Ref.	Ref.
Mutant	469 (274)	3.45 (2.60 to 4.56)	3.17 (2.38 to 4.24)	219 (136)	2.70 (1.86 to 3.92)	2.43 (1.66 to 3.55)	246 (135)	1.15 (0.63 to 2.10)	1.28 (0.69 to 2.38)
T[c]		0.89 (0.86 to 0.92)	0.89 (0.86 to 0.92)		0.93 (0.89 to 0.97)	0.93 (0.89 to 0.97)		0.94 (0.86 to 1.03)	0.93 (0.85 to 1.01)
**IHC-based TP53**									
Wildtype-like	600 (299)	Ref.	Ref.	504 (247)	Ref.	Ref.	92 (50)	Ref.	Ref.
Mutant-like	173 (93)	2.28 (1.52 to 3.42)	2.00 (1.32 to 3.03)	93 (53)	1.75 (1.01 to 3.04)	1.44 (0.82 to 2.53)	80 (40)	1.45 (0.74 to 2.82)	1.51 (0.77 to 2.96)
T[c]		0.92 (0.87 to 0.96)	0.92 (0.87 to 0.97)		0.95 (0.89 to 1.01)	0.95 (0.89 to 1.02)		0.92 (0.83 to 1.03)	0.93 (0.83 to 1.03)
**PAM50 subtype**									
Other	1,127 (606)	Ref.	Ref.	992 (520)	Ref.	Ref.	124 (79)	Ref.	Ref.
Basal-like	213 (109)	3.60 (2.52 to 5.14)	3.50 (2.43 to 5.02)	31 (17)	2.63 (1.08 to 6.41)	2.66 (1.09 to 6.52)	179 (91)	0.86 (0.54 to 1.37)	0.94 (0.58 to 1.51)
T[c]		0.85 (0.81 to 0.90)	0.85 (0.80 to 0.89)		0.90 (0.80 to 1.02)	0.91 (0.80 to 1.02)		0.97 (0.90 to 1.04)	0.97 (0.90 to 1.04)

[a]Adjusted for age at diagnosis (continuous)

[b]Additionally adjusted for tumor stage, grade, size, and node status

[c]Log of time-varying coefficient (if $T < 1$ then hazard decreases with time, and if $T > 1$ then hazard increases with time)

ER = estrogen receptor, IHC = immunohistochemistry, METABRIC = Molecular Taxonomy of Breast Cancer International Consortium, PAM50 = Prediction Analysis of Microarray 50.

Supplementary Table 4. Hazard ratio (95% confidence interval) for the association between tumor subtype and **recurrence-free survival** among breast cancer cases in **CBCS** phase 3, overall and stratified by estrogen receptor (ER) status.

	**Overall**			**ER positive**			**ER negative**		
Tumor subtype	N (events)	Minimally adjusted[a]	Adjusted[b]	N (events)	Minimally adjusted[a]	Adjusted[b]	N (events)	Minimally adjusted[a]	Adjusted[b]
**RNA-based TP53**									
Wildtype-like	797 (82)	Ref.	Ref.	764 (73)	Ref.	Ref.	33 (9)	Ref.	Ref.
Mutant-like	577 (114)	8.03 (4.38 to 14.73)	6.21 (3.27 to 11.80)	243 (51)	9.41 (4.23 to 20.90)	7.30 (3.21 to 16.63)	333 (63)	0.48 (0.13 to 1.72)	0.50 (0.13 to 1.95)
T[c]		0.61 (0.51 to 0.73)	0.61 (0.51 to 0.73)		0.64 (0.51 to 0.80)	0.64 (0.52 to 0.80)		1.20 (0.60 to 2.39)	1.17 (0.59 to 2.28)
**IHC-based TP53**									
Wildtype-like	1,260 (132)	Ref.	Ref.	1,074 (101)	Ref.	Ref.	185 (31)	Ref.	Ref.
Mutant-like	415 (64)	2.77 (1.60 to 4.79)	2.16 (1.24 to 3.78)	204 (29)	2.72 (1.19 to 6.25)	2.27 (0.98 to 5.26)	210 (35)	0.76 (0.32 to 1.77)	0.75 (0.32 to 1.76)
T[c]		0.81 (0.70 to 0.95)	0.81 (0.70 to 0.95)		0.86 (0.70 to 1.05)	0.86 (0.70 to 1.05)		1.13 (0.79 to 1.61)	1.11 (0.78 to 1.57)
**PAM50 subtype**									
Other	1,040 (127)	Ref.	Ref.	936 (108)	Ref.	Ref.	104 (19)	Ref.	Ref.
Basal-like	334 (69)	6.61 (3.68 to 11.86)	5.70 (3.11 to 10.44)	71 (16)	10.93 (3.91 to 30.57)	9.83 (3.49 to 27.64)	262 (53)	0.92 (0.37 to 2.31)	1.02 (0.40 to 2.63)
T[c]		0.58 (0.46 to 0.72)	0.58 (0.46 to 0.72)		0.53 (0.35, 0.80)	0.54 (0.36 to 0.80)		1.12 (0.72 to 1.74)	1.10 (0.71 to 1.72)

[a]Adjusted for age at diagnosis (continuous), race (Black/non-Black), and study phase

[b]Additionally adjusted for tumor stage, grade, size, and node status

[c]Log of time-varying coefficient (if $T < 1$ then hazard decreases with time, and if $T > 1$ then hazard increases with time)

CBCS = Carolina Breast Cancer Study, ER = estrogen receptor, IHC = immunohistochemistry, PAM50 = Prediction Analysis of Microarray 50.

Supplementary Table 5. Hazard ratio (95% confidence interval) for the association between tumor subtype and **recurrence-free survival** among breast cancer cases in **METABRIC**, overall and stratified by estrogen receptor (ER) status.

	**Overall**			**ER positive**			**ER negative**		
Tumor subtype	N (events)	Minimally adjusted[a]	Adjusted[b]	N (events)	Minimally adjusted[a]	Adjusted[b]	N (events)	Minimally adjusted[a]	Adjusted[b]
**RNA-based TP53**									
Wildtype-like	764 (233)	Ref.	Ref.	734 (223)	Ref.	Ref.	23 (9)	Ref.	Ref.
Mutant-like	580 (254)	3.41 (2.53 to 4.58)	2.91 (2.12 to 4.00)	293 (127)	2.87 (2.00 to 4.13)	2.47 (1.69, 3.61)	280 (123)	1.28 (0.46 to 3.60)	1.08 (0.37 to 3.16)
T[c]		0.86 (0.81 to 0.90)	0.86 (0.82 to 0.90)		0.90 (0.84 to 0.95)	0.91 (0.85, 0.96)		0.93 (0.72 to 1.20)	0.90 (0.69 to 1.18)
**DNA-based TP53**									
Wildtype	838 (280)	Ref.	Ref.	774 (249)	Ref.	Ref.	54 (27)	Ref.	Ref.
Mutant	470 (200)	2.41 (1.81 to 3.22)	2.08 (1.54 to 2.81)	220 (95)	2.08 (1.42 to 3.05)	1.78 (1.21, 2.63)	246 (104)	0.95 (0.52 to 1.72)	1.02 (0.55 to 1.89)
T[c]		0.89 (0.84 to 0.93)	0.89 (0.84 to 0.93)		0.95 (0.89 to 1.01)	0.95 (0.89, 1.01)		0.94 (0.82 to 1.07)	0.92 (0.80 to 1.05)
**IHC-based TP53**									
Wildtype-like	601 (203)	Ref.	Ref.	505 (161)	Ref.	Ref.	92 (41)	Ref.	Ref.
Mutant-like	173 (61)	1.67 (1.07 to 2.61)	1.42 (0.90 to 2.24)	93 (35)	1.58 (0.88 to 2.84)	1.23 (0.67, 2.25)	80 (26)	1.12 (0.51 to 2.44)	1.20 (0.55 to 2.66)
T[c]		0.90 (0.83 to 0.98)	0.90 (0.83 to 0.98)		0.95 (0.86 to 1.04)	0.95 (0.87, 1.05)		0.86 (0.69 to 1.08)	0.86 (0.69 to 1.08)
**PAM50 subtype**									
Other	1,128 (402)	Ref.	Ref.	993 (337)	Ref.	Ref.	124 (62)	Ref.	Ref.
Basal-like	213 (83)	2.68 (1.84 to 3.90)	2.39 (1.63 to 3.50)	31 (11)	2.74 (1.05 to 7.16)	2.71 (1.05, 6.98)	179 (70)	0.97 (0.59 to 1.58)	1.06 (0.64 to 1.75)
T[c]		0.80 (0.73 to 0.88)	0.80 (0.73 to 0.88)		0.81 (0.65 to 1.03)	0.82 (0.65, 1.02)		0.91 (0.81 to 1.02)	0.91 (0.81 to 1.02)

[a]Adjusted for age at diagnosis (continuous)

[b]Additionally adjusted for tumor stage, grade, size, and node status

[c]Log of time-varying coefficient (if $T < 1$ then hazard decreases with time, and if $T > 1$ then hazard increases with time)

ER = estrogen receptor, IHC = immunohistochemistry, METABRIC = Molecular Taxonomy of Breast Cancer International Consortium, PAM50 = Prediction Analysis of Microarray 50.

Supplementary Table 6. Likelihood ratio test (χ^2 test statistic [p-value]) of the addition of each tumor marker in the fully adjusted prognostic model, by study population and estrogen receptor status.

	CBCS			METABRIC		
**Tumor marker**	Overall	ER positive	ER negative	Overall	ER positive	ER negative
TP53 subtype (RNA)	45.7 (<0.001)	10.5 (0.005)	12.5 (0.002)	57.6 (<0.001)	24.7 (<0.001)	1.9 (0.379)
TP53 subtype (DNA)	16.5 (<0.001)	9.9 (0.007)	2.3 (0.313)	40.6 (<0.001)	18.5 (<0.001)	2.9 (0.236)
TP53 subtype (IHC)	1.3 (0.515)	5.8 (0.056)	0.7 (0.722)	11.1 (0.004)	4.1 (0.126)	3.0 (0.222)
Basal-like subtype	24.3 (<0.001)	3.6 (0.166)	5.5 (0.065)	39.2 (<0.001)	5.9 (0.052)	7.5 (0.023)

CBCS = Carolina Breast Cancer Study, ER = estrogen receptor, IHC = immunohistochemistry, METABRIC = Molecular Taxonomy of Breast Cancer International Consortium.

Supplementary Figure 1. Study population flowchart for CBCS and METABRIC.

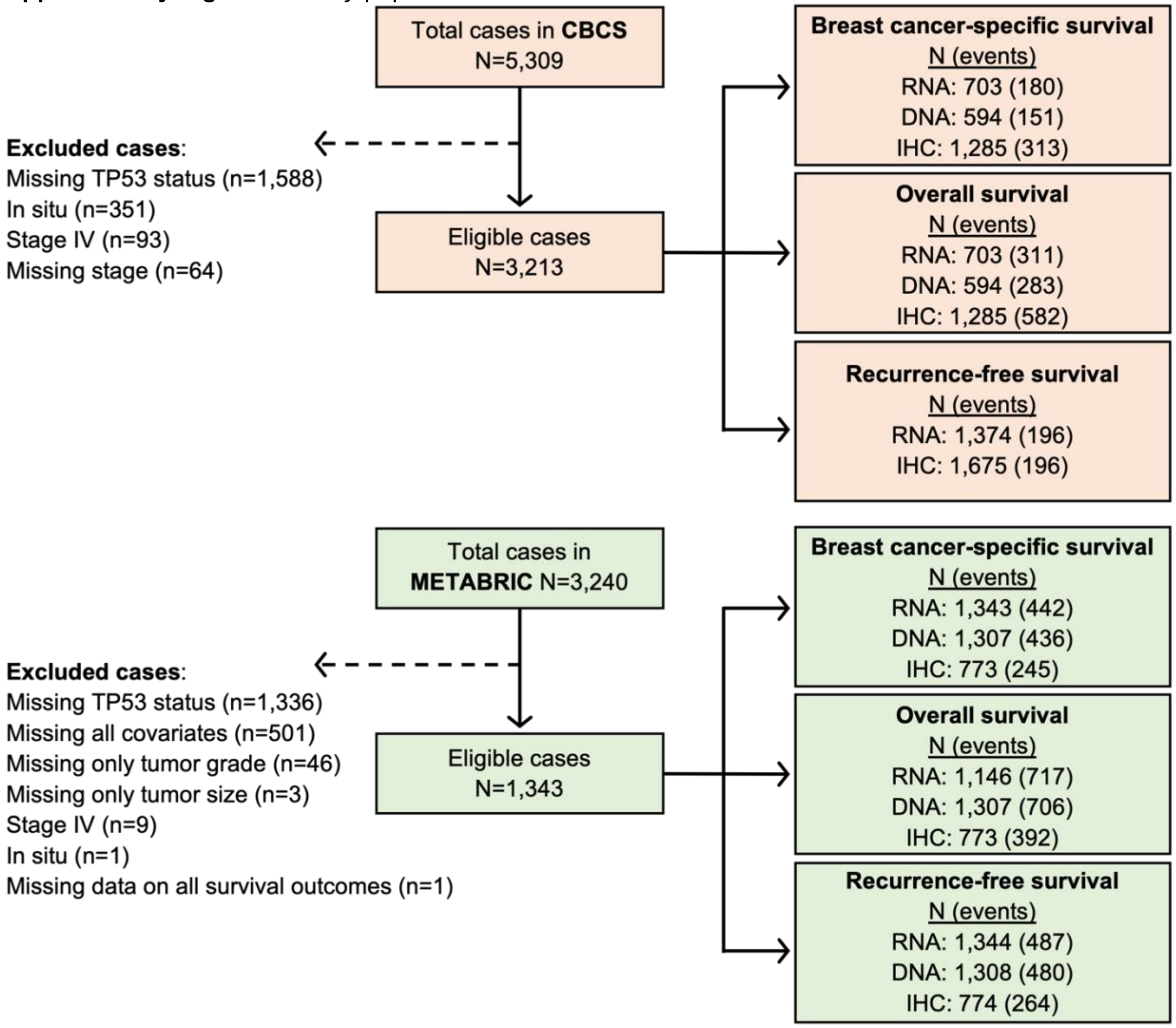

CBCS = Carolina Breast Cancer Study, IHC = immunohistochemistry, METABRIC = Molecular Taxonomy of Breast Cancer International Consortium.

Supplementary Figure 2. Kaplan-Meier survival curves for **overall survival** by tumor subtype, overall and stratified by ER status, among node negative breast cancer cases in **CBCS**.

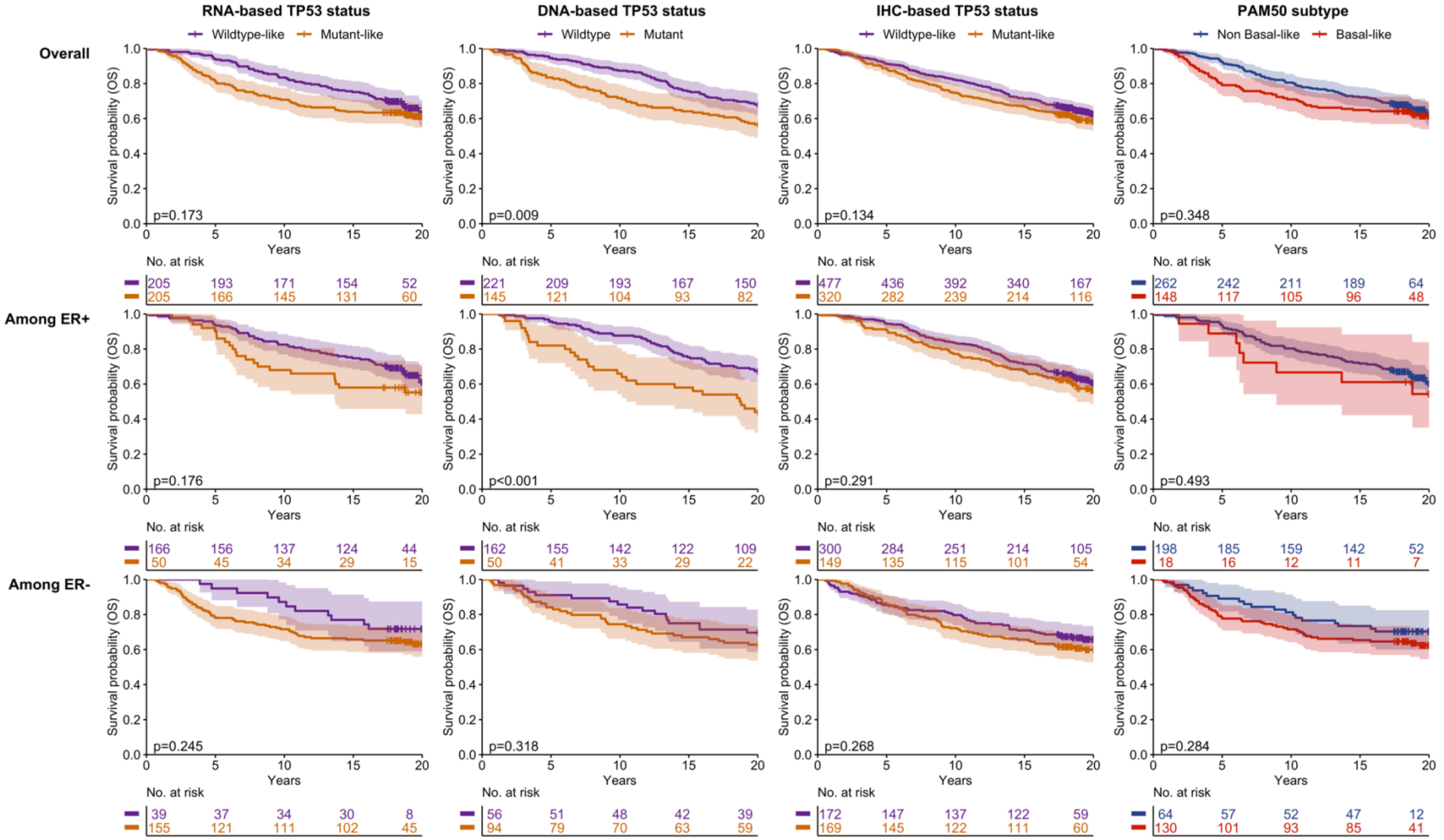

p-values correspond to the log-rank test. The shaded regions correspond to the 95% confidence interval.

CBCS = Carolina Breast Cancer Study, ER = estrogen receptor, IHC = immunohistochemistry, OS = overall survival, PAM50 = Prediction Analysis of Microarray 50.

Supplementary Figure 3. Kaplan-Meier survival curves for **overall survival** by tumor subtype, overall and stratified by ER status, among node negative breast cancer cases in **METABRIC**.

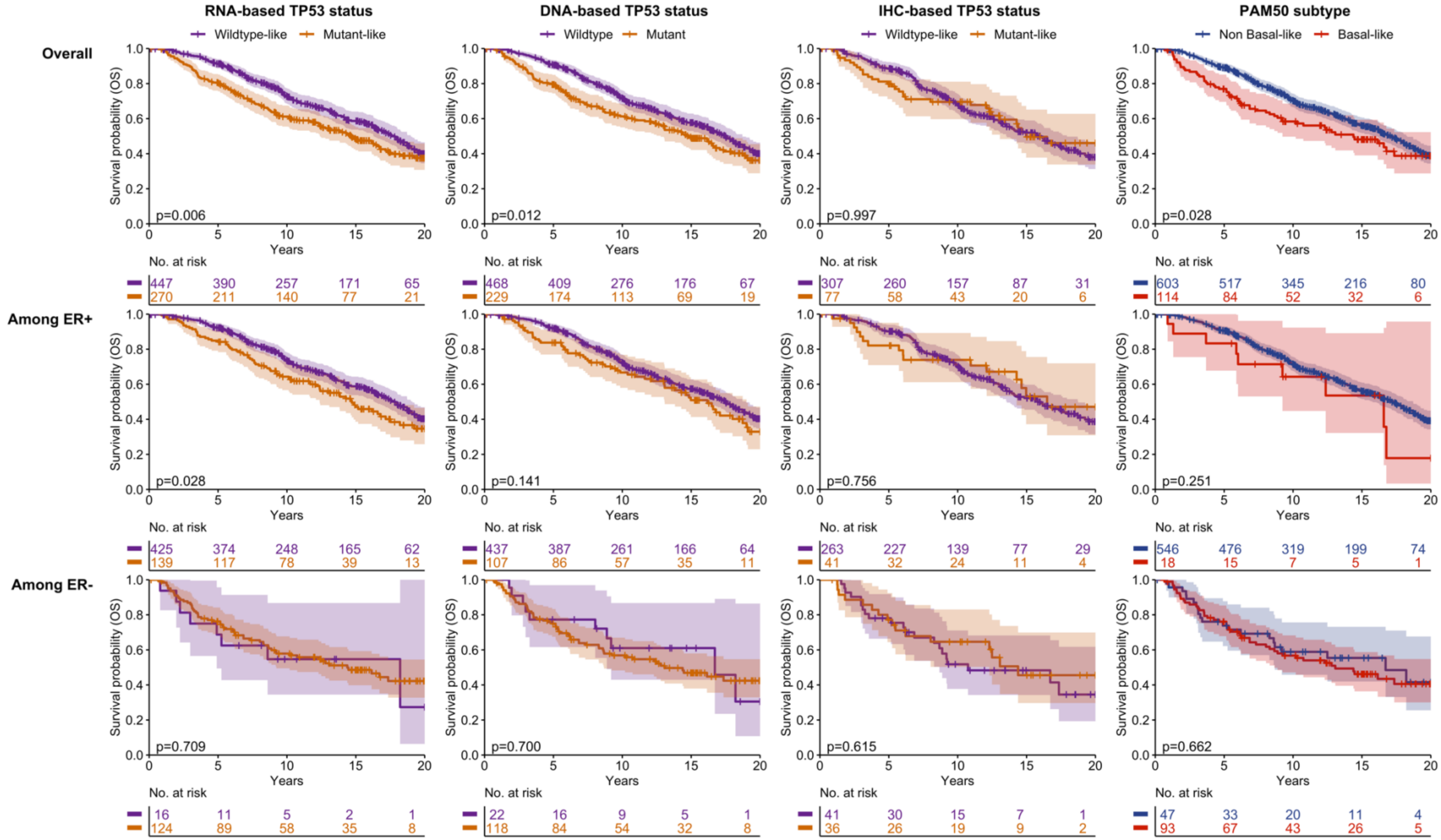

p-values correspond to the log-rank test. The shaded regions correspond to the 95% confidence interval.

ER = estrogen receptor, IHC = immunohistochemistry, METABRIC = Molecular Taxonomy of Breast Cancer International Consortium, OS = overall survival, PAM50 = Prediction Analysis of Microarray 50.

Supplementary Figure 4. Association between tumor subtype and **overall survival** among breast cancer cases in CBCS and METABRIC, overall and stratified by estrogen receptor (ER) status.

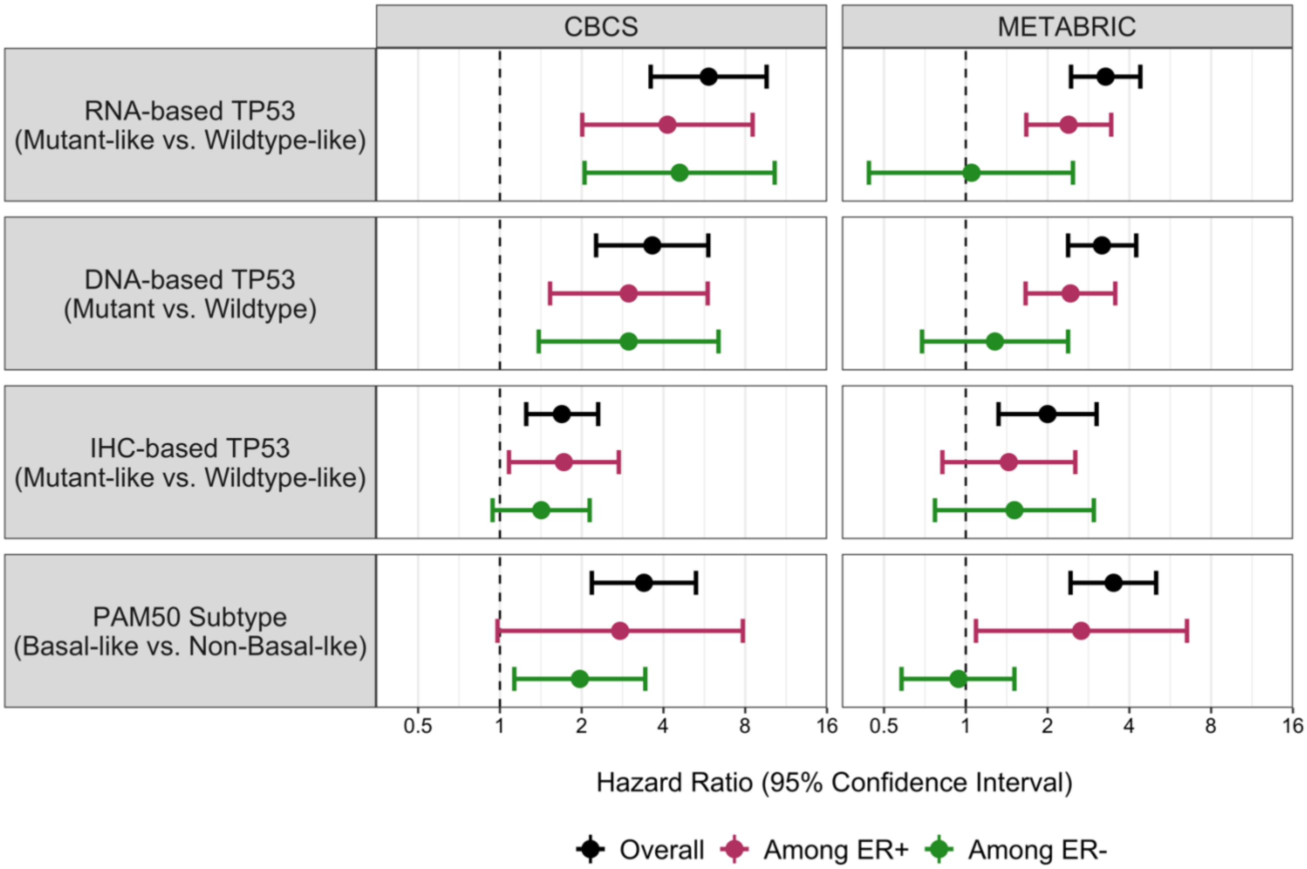

The error bars correspond to the 95% confidence intervals.
CBCS = Carolina Breast Cancer Study, ER = estrogen receptor, IHC = immunohistochemistry, METABRIC = Molecular Taxonomy of Breast Cancer International Consortium, PAM50 = Prediction Analysis of Microarray 50.

Supplementary Figure 5. Kaplan-Meier survival curves for **recurrence-free survival** by tumor subtype, overall and stratified by ER status, among node negative breast cancer cases in **CBCS**.

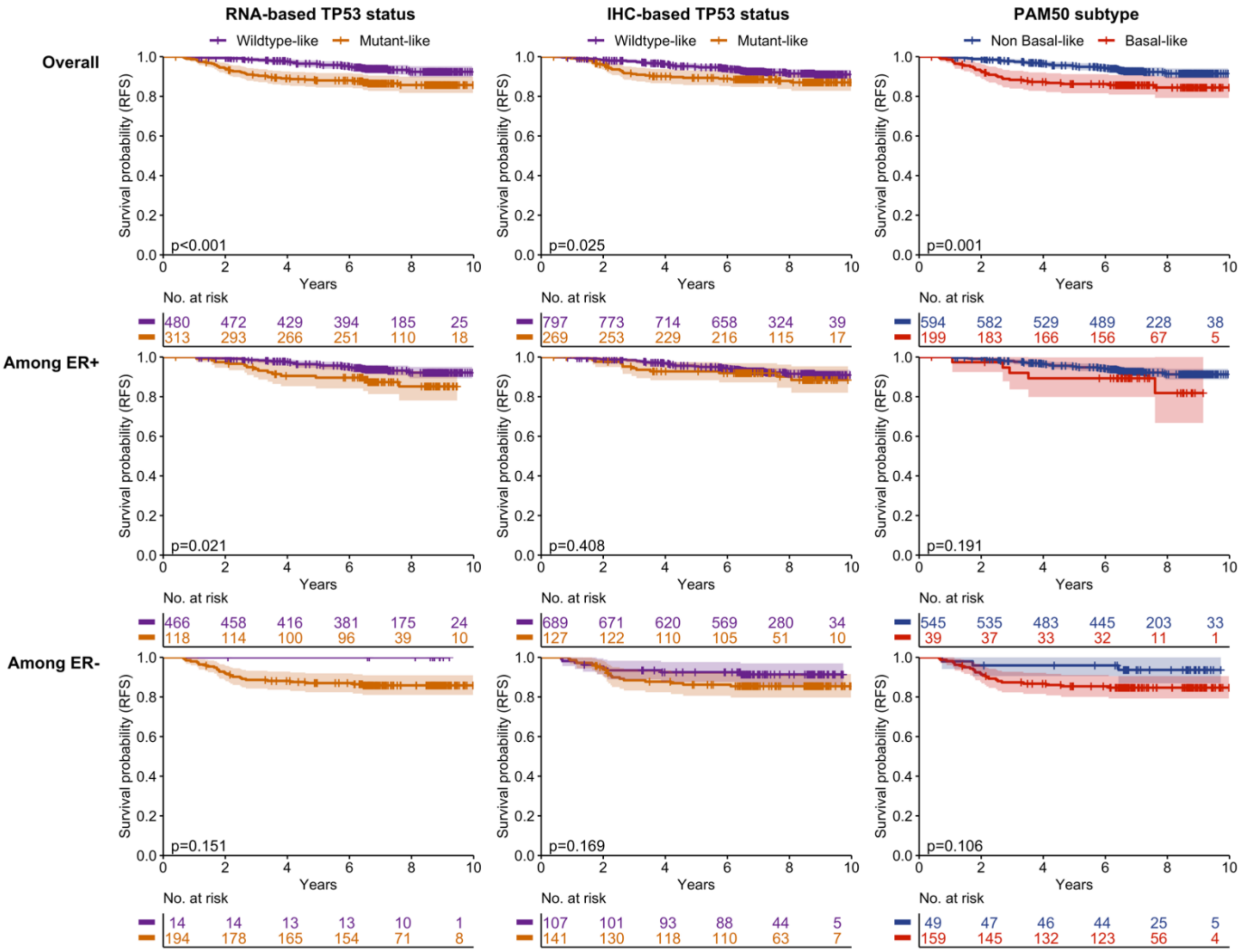

p-values correspond to the log-rank test. The shaded regions correspond to the 95% confidence interval.
CBCS = Carolina Breast Cancer Study, ER = estrogen receptor, IHC = immunohistochemistry, PAM50 = Prediction Analysis of Microarray 50, RFS = recurrence-free survival.

Supplementary Figure 6. Kaplan-Meier survival curves for **recurrence-free survival** by tumor subtype, overall and stratified by ER status, among node negative breast cancer cases in **METABRIC.**

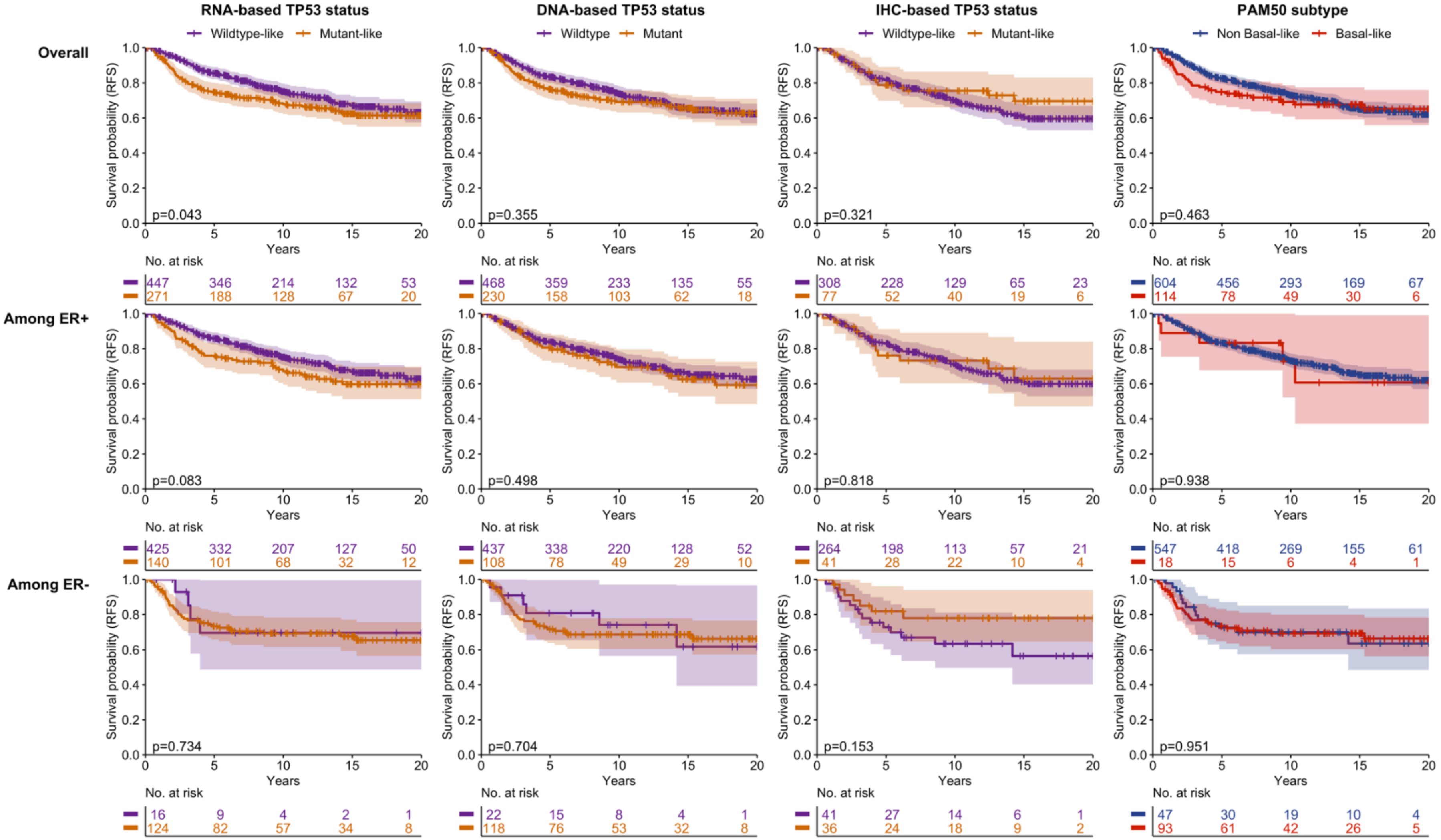

p-values correspond to the log-rank test. The shaded regions correspond to the 95% confidence interval.

ER = estrogen receptor, IHC = immunohistochemistry, METABRIC = Molecular Taxonomy of Breast Cancer International Consortium, PAM50 = Prediction Analysis of Microarray 50, RFS = recurrence-free survival.

Supplementary Figure 7. Association between tumor subtype and **recurrence-free survival** among breast cancer cases in CBCS and METABRIC, overall and stratified by estrogen receptor (ER) status.

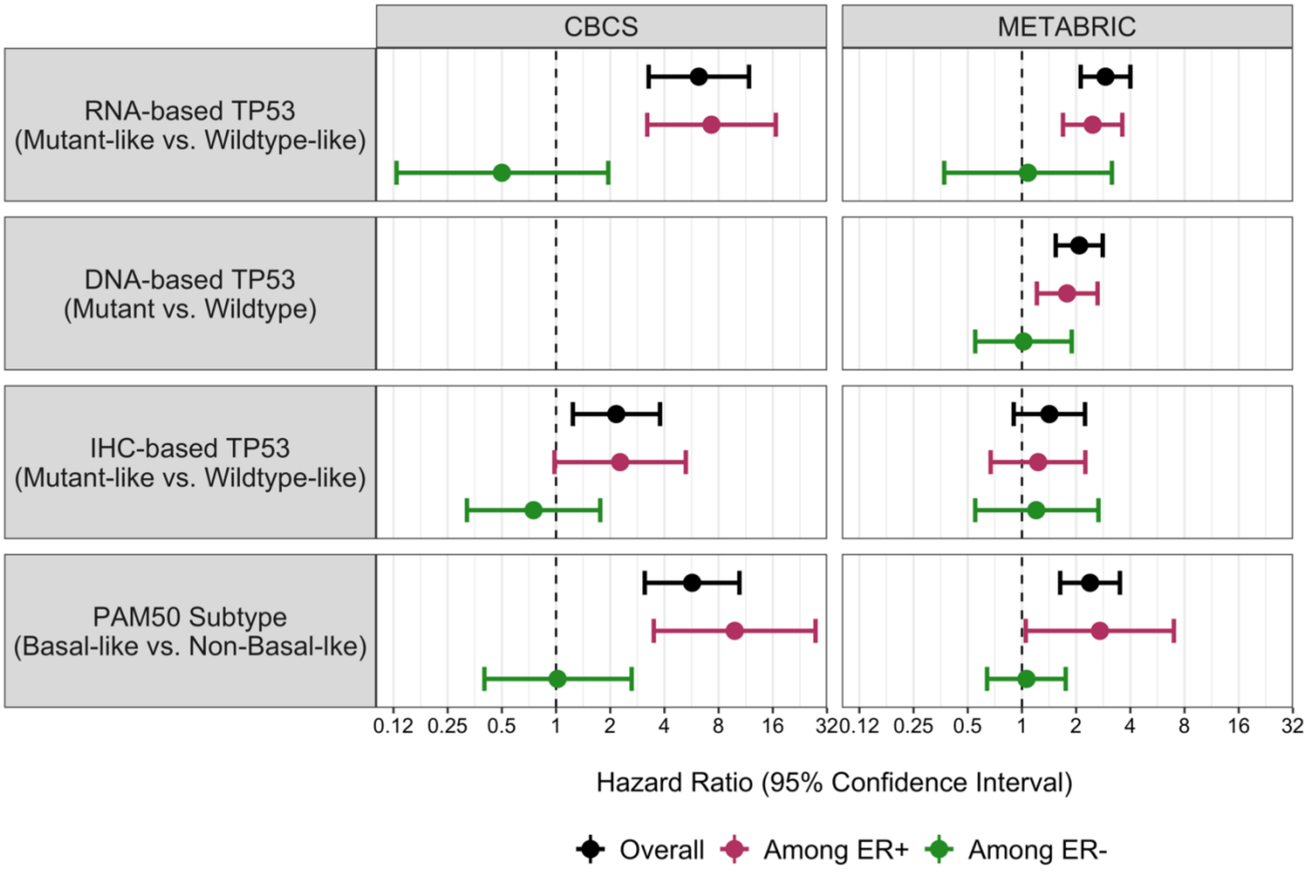

The error bars correspond to the 95% confidence intervals.
CBCS = Carolina Breast Cancer Study, ER = estrogen receptor, IHC = immunohistochemistry, METABRIC = Molecular Taxonomy of Breast Cancer International Consortium, PAM50 = Prediction Analysis of Microarray 50.
