# Supplementary material for: Prognostic significance of RNA-based TP53 pathway function among estrogen receptor positive and negative breast cancer cases
Source: NPJ Breast Cancer. 2022 Jun 14;8:74. doi: 10.1038/s41523-022-00437-7 (PMC9198049; doi:10.1038/s41523-022-00437-7)
Supplement: Supplementary file 2 — Reporting Summary [file 41523_2022_437_MOESM2_ESM.pdf]

nature portfolio

Corresponding author(s): Melissa Troester

Last updated by author(s): Feb 23, 2022

Reporting Summary

Nature Portfolio wishes to improve the reproducibility of the work that we publish. This form provides structure for consistency and transparency in reporting. For further information on Nature Portfolio policies, see our Editorial Policies and the Editorial Policy Checklist.

Statistics

For all statistical analyses, confirm that the following items are present in the figure legend, table legend, main text, or Methods section.

n/a	Confirmed	
☒	☐	The exact sample size (*n*) for each experimental group/condition, given as a discrete number and unit of measurement
☐	☒	A statement on whether measurements were taken from distinct samples or whether the same sample was measured repeatedly
☐	☒	The statistical test(s) used AND whether they are one- or two-sided<br>*Only common tests should be described solely by name; describe more complex techniques in the Methods section.*
☐	☒	A description of all covariates tested
☐	☒	A description of any assumptions or corrections, such as tests of normality and adjustment for multiple comparisons
☐	☒	A full description of the statistical parameters including central tendency (e.g. means) or other basic estimates (e.g. regression coefficient) AND variation (e.g. standard deviation) or associated estimates of uncertainty (e.g. confidence intervals)
☐	☒	For null hypothesis testing, the test statistic (e.g. *F*, *t*, *r*) with confidence intervals, effect sizes, degrees of freedom and *P* value noted<br>*Give P values as exact values whenever suitable.*
☒	☐	For Bayesian analysis, information on the choice of priors and Markov chain Monte Carlo settings
☒	☐	For hierarchical and complex designs, identification of the appropriate level for tests and full reporting of outcomes
☐	☒	Estimates of effect sizes (e.g. Cohen's *d*, Pearson's *r*), indicating how they were calculated

Our web collection on statistics for biologists contains articles on many of the points above.

Software and code

Policy information about availability of computer code

Data collection	N/A
Data analysis	Statistical analyses were conducted in R software version 4.0.2 (R Foundation for Statistical Computing).

For manuscripts utilizing custom algorithms or software that are central to the research but not yet described in published literature, software must be made available to editors and reviewers. We strongly encourage code deposition in a community repository (e.g. GitHub). See the Nature Portfolio guidelines for submitting code & software for further information.

Data

Policy information about availability of data

All manuscripts must include a data availability statement. This statement should provide the following information, where applicable:

- Accession codes, unique identifiers, or web links for publicly available datasets
- A description of any restrictions on data availability
- For clinical datasets or third party data, please ensure that the statement adheres to our policy

The CBCS datasets generated and/or analyzed during the current study are not publicly available due to some human subjects restrictions, but may be available from the corresponding author on reasonable request. METABRIC data can be found here: http://www.cbioportal.org/study?id=brca_metabric.

Field-specific reporting

Please select the one below that is the best fit for your research. If you are not sure, read the appropriate sections before making your selection.

☒ Life sciences ☐ Behavioural & social sciences ☐ Ecological, evolutionary & environmental sciences

For a reference copy of the document with all sections, see nature.com/documents/nr-reporting-summary-flat.pdf

Life sciences study design

All studies must disclose on these points even when the disclosure is negative.

Sample size	The sample size was determined by the size of the existing cohorts.
Data exclusions	All exclusions were pre-established and based on scientific considerations. Cases missing TP53 status or stage information, as well as those diagnosed with in situ or stage IV disease were excluded.
Replication	The Carolina Breast Cancer Study does random reviews of clinical records, data extractions, and biological analysis to confirm reproducibility.
Randomization	This was an observational study, not a clinical trial. Therefore, randomization was not applied.
Blinding	This was an observational study, not a clinical trial. Therefore, blinding was not applied.

Reporting for specific materials, systems and methods

We require information from authors about some types of materials, experimental systems and methods used in many studies. Here, indicate whether each material, system or method listed is relevant to your study. If you are not sure if a list item applies to your research, read the appropriate section before selecting a response.

Materials & experimental systems

n/a	Involved in the study	
☒	☐	Antibodies
☒	☐	Eukaryotic cell lines
☒	☐	Palaeontology and archaeology
☒	☐	Animals and other organisms
☐	☒	Human research participants
☒	☐	Clinical data
☒	☐	Dual use research of concern

Methods

n/a	Involved in the study	
☒	☐	ChIP-seq
☒	☐	Flow cytometry
☒	☐	MRI-based neuroimaging

Human research participants

Policy information about studies involving human research participants

Population characteristics	CBCS cases include women between 23-74 years of age diagnosed with invasive breast cancer. METABRIC cases include women between 22-96 years of age diagnosed with invasive breast cancer.
Recruitment	CBCS participants were enrolled in three phases between 1993 and 2013 from 44 counties in central and eastern North Carolina. Incident cases were identified using a rapid case ascertainment system through the NC Central Cancer Registry. Eligible cases included women aged 20-74 years, living within the study area, with a new diagnosis of a first primary invasive breast cancer.<br><br>METABRIC includes fresh-frozen primary breast tumors collected from five tumor banks across UK and Canada between 1977 and 2005.
Ethics oversight	All CBCS study procedures were approved by the University of North Carolina School of Medicine Institutional Review Board.

Note that full information on the approval of the study protocol must also be provided in the manuscript.
